# Effects of home confinement on physical activity, nutrition, and sleep quality during the COVID-19 outbreak in amateur and elite athletes

**DOI:** 10.3389/fnut.2023.1143340

**Published:** 2023-04-13

**Authors:** Morteza Taheri, Kadijeh Irandoust, Luis Felipe Reynoso-Sánchez, Hussein Muñoz-Helú, Karla Noelia Cruz-Morales, Raymundo Torres-Ramírez, Masoud Mirmoezzi, Leila Youzbashi, Fatemeh Mirakhori, Ismail Dergaa, Rodrigo Luiz Vancini, Leonardo Jose Mataruna-Dos-Santos, Diana Korinna Zazueta-Beltrán, Hassane Zouhal, Roxana Abril Morales-Beltrán, Yusuf Soylu, Amairani Molgado-Sifuentes, Juan González-Hernández, Germán Hernández-Cruz, Luis Bernardo Bojorquez Castro, Cem Kurt, Khaled Trabelsi, Hamdi Chtourou, Ali Seghatoleslami, Manuel Octavio López-Camacho, Ramón Ernesto Mendoza-Baldenebro, Farnaz Torabi, Helmi Ben Saad, Jad Adrian Washif, Jana Strahler, Andressa Fontes Guimarães-Mataruna, Tyler W. Lebaron, Ebrahim Shaabani Ezdini, Marjan Sadat Rezaei, Mozhgan Moshtagh, Fatma Hilal Yagin, Mehmet Gülü, Atefeh Esmaeili, Patrick Müller, Achraf Ammar, Egeria Scoditti, Sergio Garbarino, Luca Puce, Nicola Luigi Bragazzi, Hadi Nobari

**Affiliations:** ^1^Department of Sport Sciences, Imam Khomeini International University, Qazvin, Iran; ^2^Department of Motor Behavior, Faculty of Sport Sciences and Health, University of Tehran, Tehran, Iran; ^3^Institute of Future Studies, Imam Khomeini International University, Qazvin, Iran; ^4^Department of Social Sciences and Humanities, Autonomous University of Occident, Los Mochis, Mexico; ^5^Department of Economic-Administrative Sciences, Autonomous University of Occident, Los Mochis, Mexico; ^6^Faculty of Physical Education and Sport Science, Islamic Azad University, Tehran, Iran; ^7^Department of sport sciences, Faculty of Humanities, University of Zanjan, Zanjan, Iran; ^8^Primary Health Care Corporation, Doha, Qatar; ^9^Research Unit Physical Activity, Sport, and Health, (UR18JS01), National Observatory of Sport, Tunis, Tunisia; ^10^High Institute of Sport and Physical Education, University of Sfax, Sfax, Tunisia; ^11^Centro de Educação Física e Desportos, Universidade Federal do Espírito Santo, Vitória, Espírito Santo, Brazil; ^12^Sport Management Department, Faculty of Management, Canadian University Dubai, Dubai, United Arab Emirates; ^13^Univ Rennes, M2S (Laboratoire Mouvement, Sport, Santé) - EA 1274, Rennes, France; ^14^Institute International des Sciences du Sport, Irodouer, France; ^15^Faculty of Sport Sciences, Tokat Gaziosmanpasa University, Tokat, Türkiye; ^16^Faculty of Nutrition Sciences and Gastronomy, Autonomous University of Sinaloa, Culiacán, Mexico; ^17^Department of Personality, Evaluation and Psychological Treatment, University of Granada, Granada, Spain; ^18^Faculty of Sport Organization, Autonomous University of Nuevo Leon, San Nicolás de los Garza, Mexico; ^19^Department of Social Sciences and Humanities, Autonomous University of Occident, Guasave, Mexico; ^20^Kirkpinar Sport Sciences Faculty, Trakya University, Edirne, Türkiye; ^21^Faculty of Sports Science, University of Birjand, Birjand, Iran; ^22^Department of Physical Education, Payame Noor University, Tehran, Iran; ^23^Faculty of Medicine, Farhat Hached Hospital, Research Laboratory “Heart Failure, LR12SP09”, University of Sousse, Sousse, Tunisia; ^24^Sports Performance Division, Institut Sukan Negara Malaysia (National Sports Institute of Malaysia), Kuala Lumpur, Malaysia; ^25^Sport Psychology, Institute of Sport and Sport Science, University of Freiburg, Freiburg, Germany; ^26^Department of Communication and Arts, University of Beira Interior, Covilha, Portugal; ^27^Department of Kinesiology and Outdoor Recreation, Southern Utah University, Cedar City, UT, United States; ^28^Molecular Hydrogen Institute, Enoch, UT, United States; ^29^Centre of Experimental Medicine, Institute for Heart Research, Slovak Academy of Sciences, Bratislava, Slovakia; ^30^Social Determinants of Health Research Center, Faculty of Health, Birjand University of Medical Sciences, Birjand, Iran; ^31^Department of Biostatistics, and Medical Informatics, Faculty of Medicine, Inonu University, Malatya, Türkiye; ^32^Department of Sport Management, Faculty of Sport Sciences, Kirikkale University, Kirikkale, Türkiye; ^33^Division of Cardiology and Angiology, German Center for Neurodegenerative Diseases (DZNE), Univerisity Hospital Magdeburg, Magdeburg, Germany; ^34^Department of Training and Movement Science, Institute of Sport Science, Johannes Gutenberg-University Mainz, Mainz, Germany; ^35^Interdisciplinary Laboratory in Neurosciences, Physiology and Psychology: Physical Activity, Health and Learning (LINP2), UFR STAPS (Faculty of Sport Sciences), UPL, Paris Nanterre University, Nanterre, France; ^36^National Research Council (CNR)-Institute of Clinical Physiology (IFC), Lecce, Italy; ^37^Department of Neuroscience, Rehabilitation, Ophthalmology, Genetics, Maternal and Child Health (DINOGMI), University of Genoa, Genoa, Italy; ^38^Human Nutrition Unit, Department of Food and Drugs, Medical School, University of Parma, Parma, Italy; ^39^Faculty of Sport Sciences, University of Extremadura, Cáceres, Spain

**Keywords:** physical activity, eating behaviors, lifestyle behaviors, sleep quality, COVID-19

## Abstract

**Introduction:**

Despite the progress in the management of the pandemic caused by COVID-19, it is necessary to continue exploring and explaining how this situation affected the athlete population around the world to improve their circumstances and reduce the negative impact of changes in their lifestyle conditions that were necessitated due to the pandemic. The aim of this study was to analyze the moderating influence of physical activity (PA) and dietary habits on the impact of the COVID-19 pandemic experience on sleep quality in elite and amateur athletes.

**Materials and methods:**

A total of 1,420 elite (40.1%) and amateur (59.9%) athletes (41% women; 59% men) from 14 different countries participated in a cross-sectional design study. Data were collected using a battery of questionnaires that identified sociodemographic data, sleep quality index, PA levels, dietary habits, and the athletes' perception of their experience during the COVID-19 pandemic. Means and standard deviations were calculated for each variable. The analysis of variances and the correlation between variables were carried out with non-parametric statistics. A simple moderation effect was calculated to analyze the interaction between PA or dietary habits on the perception of the COVID-19 experience effect on sleep quality in elite and amateur athletes.

**Results:**

The PA level of elite athletes was higher than amateur athletes during COVID-19 (*p* < 0.001). However, the PA level of both categories of athletes was lower during COVID-19 than pre-COVID-19 (*p* < 0.01). In addition, amateurs had a higher diet quality than elite athletes during the pandemic (*p* = 0.014). The perception of the COVID-19 experience as controllable was significantly higher (*p* = 0.020) among elite athletes. In addition, two moderating effects had significant interactions. For amateur athletes, the PA level moderated the effect of controllable COVID-19 experience on sleep quality [*F*_(3,777)_ = 3.05; *p* = 0.028], while for elite athletes, the same effect was moderated by dietary habits [*F*_(3,506)_ = 4.47, *p* = 0.004].

**Conclusion:**

Elite athletes had different lifestyle behaviors compared to amateurs during the COVID-19 lockdown. Furthermore, the relevance of maintaining high levels of PA for amateurs and good quality dietary habits by elite athletes was noted by the moderating effect that both variables had on the influence of the controllable experience during the COVID-19 pandemic on sleep quality.

## Introduction

The confinement brought about by the COVID-19 pandemic caused changes in different areas of society, affecting the economic and health spheres, as well as interpersonal relationships (1–3). Several sources have pointed out that the restrictions on coexistence imposed by the health authorities led to the closure of public and private spaces for physical exercise, increasing sedentary lifestyles in the population that were deprived of their places for exercising and playing sports (4, 5). Under these circumstances, isolation caused changes in lifestyles which both amateur and elite athletes had to adapt to. Disorders related to sleep, dietary habits, and changes in physical activity (PA) levels (6, 7) were among the main problems reported.

A consequence of the confinement and virtuality to which the population had to become accustomed to was an increase in the development and use of various digital platforms, applications, and gadgets as support for the practice of physical exercise and even to monitor certain capabilities and psychophysiological responses generated by the stimulus of training performed by athletes and non-athletes (6). The use of these methods and tools was employed by coaches and institutions as an alternative for the promotion and execution of PA and sport (8). However, it was found that coaches had to adapt their training sessions to the conditions of each sport (9).

Changes in the planning were primarily oriented toward the maintenance of the general physical aptitudes of the athletes and in a very small percentage to reinforce the special requirements of each sport. This resulted in a decrease in athlete motivation, which is an important factor in their performance (10, 11). Despite the above findings, studies such as the one by Washif et al. (11) reported that athletes with higher competitive levels suffered less affectation to maintain their training routines with respect to lower-level athletes, even when they carried it out in total isolation without sporting equipment and without physical accompaniment as commonly performed.

Due to the modifications in the athletes' training routine during the pandemic, the literature reported changes in their sleep habits, reduced hours and quality of sleep, and even insomnia (12, 13). The decrease in sleep time and the acquisition of unhealthy habits such as prolonged naps and increased consumption of caffeine, alcohol, and tobacco were some of the behaviors that athletes picked up during the pandemic period (13). However, as previously known, exposure to the lights from cellphone screens late at night affected sleep latency (14, 15), altering not only schedules but also causing problems related to mental health. One possible alternative recommended by specialists was that athletes and coaches consider sleep habits and chronobiology to adapt PA programs that favor them, especially in those individuals who generally sleep in evening shifts (14) and thereby seek a faster adaptation to traditional training schedules once out of the pandemic (16).

In addition to alterations in the frequency and intensity of physical exercise and sleep habits, there is scientific evidence that indicates that athletes presented disorders in their dietary habits. This was mostly due to anxiety problems caused by isolation and the acquisition of leisure activities, such as spending more hours in front of electronic devices accompanied by the consumption of unhealthy foods (17–19) and less control or follow-up by their multidisciplinary team, together with the relaxation generated by the cancellation or changes in competitions and training programs (20). Nutrition is one of the most important factors for athletes, where the quality, quantity, and time of intake should be balanced according to the recommendations of nutrition experts to support the maintenance of optimal athletic performance (4).

Throughout the COVID-19 pandemic, many researchers have identified how the coping capacity and lived experiences of elite and amateur athletes have had different repercussions on their physical and mental health, as well as alterations in sleep and nutrition during confinement (21, 22). Some authors have shown that athletes tended to live the experiences associated with the pandemic in different ways, with the increase in anxiety and stress levels being most reported, especially in amateur athletes (23), mainly due to the fear of being infected (24, 25). In contrast, in the case of high-performance athletes, although stress was present, coping with it was more controlled, since their training habits and participation in competitions allowed them to develop resilience skills (23, 26). Furthermore, it has been suggested that one strategy to help diminish the negative effects linked to the pandemic was the practice of physical exercise (27); therefore, for athletes, maintaining the levels of their training routine influenced the maintenance of a positive psychological state (11, 14, 28).

According to the literature, some investigations have reported changes in the athletes' behavior during the pandemic period, such as modifications in their training practice, PA levels, diet, and sleep habits, as well as mental health and its possible relationship with the mentioned variables. Despite the above findings, it is necessary to deepen our understanding of the specificities that athletes have suffered according to their sports level, as well as identify the elements that can help ameliorate the consequences of this pandemic or similar situations in the future. Thus, the present study aimed to analyze the moderating influence of the PA level and dietary habits over the COVID-19 pandemic experience effect on sleep quality in elite and amateur athletes. In accordance with the existing theoretical foundation, the authors of the study set out the following hypotheses: (i) elite athletes, compared to amateur level athletes, exhibit (a) higher levels of PA performed during the pandemic; (b) better sleep quality; (c) better dietary habits; (d) and had a more positive COVID-19 pandemic experience; (ii) the perception of negative experiences lived during the COVID-19 pandemic have a negative relationship with the levels of PA, sleep quality, and dietary habits, while positive experiences have a positive relationship with the indicated variables; (iii) the level of PA practiced moderates the effect of the experience during the COVID-19 pandemic on sleep quality; (iv) dietary habits moderate the effect of the experience during the COVID-19 pandemic on sleep quality.

## Materials and methods

### Study design

The study was cross-sectional and designed to compare the long-term effect of the situational experience of the coronavirus pandemic crisis on the sleep quality, PA level, and dietary habits of elite and amateur athletes from four continents.

### Participants

The sample was recruited by a non-probabilistic snowball sampling method. A total of 1,702 athletes were invited to participate in our study (response rate = 83.43%). The final sample had 1,420 respondents (elite athletes 40.1%, *e.g*., competed at the international or national level, and amateur athletes 59.9%, *e.g*., competed at the local or recreational level) who completed the survey. Participants were drawn from 14 different countries from four continents: Africa (Tunisia, Algeria, and Egypt), America (United States of America, Mexico, and Brazil), Asia (Turkey, United Arab Emirates, Pakistan, and Iran), and Europe (Portugal, England, Lithuania, Spain, Germany, and France). Concerning gender distribution, 41% of the participants were women and 59% were men. To be part of the study, athletes had to meet the following inclusion criteria: a) to be 12 years of age or older at the time of the study; b) have been confined for at least 1 week or more during the pandemic; and c) not having missed more than 1 week of training due to injury or illness. The individual characteristics of the participants are provided in Table 1.

**Table 1:** Descriptive statistics of individual characteristics.

**Demographics**	**Amateur *n* (%)**	**Elite *n* (%)**	***P*-value**
**Sport discipline**			
Ball sports (soccer, basketball, handball, volleyball, tennis)	175 (12.3)	235 (16.5)	<0.001^a^
Athletics (track and field, triathlon, archery, cycling)	78 (5.5)	66 (4.6)	<0.001^a^
Strength (weightlifting, bodybuilding, physical fitness)	316 (22.3)	125 (8.8)	<0.001^a^
Aquatics (swimming, waterpolo, diving, rowing)	137 (9.6)	85 (6.0)	<0.001^a^
Combat sports (boxing, taekwondo, karate, judo)	109 (7.8)	54 (3.8)	<0.001^a^
Other	36 (2.5)	4 (0.3)	<0.001^a^
**Marital status**			
Married	166 (11.7)	116 (8.2)	<0.001^a^
Single	632 (44.5)	391 (27.5)	<0.001^a^
Solid partnership	41(2.9)	60 (4.2)	<0.001^a^
Divorced	12 (0.9)	2 (0.1)	<0.001^a^
**Count of households**			
I live alone	295 (20.8)	193 (13.6)	<0.001^a^
Live 2	88 (6.2)	71 (5.0)	<0.001^a^
More than 2	468 (33.0)	305 (21.4)	<0.001^a^
**History of COVID-19**			
Yes	257 (18.1)	219 (15.4)	<0.001^a^
No	594 (41.8)	350 (24.7)	<0.001^a^
**Getting vaccinated**			
Yes	812 (57.2)	527 (37.1)	<0.001^a^
No	39 (2.8)	42 (2.9)	0.025^a^
**Financially secure**			
Yes	270 (19.0)	203 (14.3)	<0.001^a^
No	581 (40.9)	366 (28.8)	<0.001^a^
**Weeks spent social distancing**			
1–4 weeks	462 (32.6)	366 (25.8)	<0.001^a^
1–2 months	179 (12.6)	101 (7.1)	<0.001^a^
2–4 months	121 (8.5)	73 (5.1)	<0.001^a^
More than 4 months	89 (6.3)	29 (2.0)	<0.001^a^
**Smoking**			
Daily	21 (1.5)	27 (1.9)	0.033^a^
Occasionally	41 (2.9)	22 (1.6)	<0.001^a^
Never	789 (55.6)	520 (36.5)	<0.001^a^
**Mental support before COVID-19**			
Yes	24 (1.7)	9 (0.6)	<0.001^a^
No	827 (58.3)	560 (39.4)	<0.001^a^
**Mental support during COVID-19**			
Yes	102 (7.1)	72 (5.1)	<0.001^a^
No	749 (52.8)	497 (35.0)	<0.001^a^

^a^Chi-square test.

### Data collection tools

An online survey divided into five sections was applied. Sections 1 to 3 consisted of *ad hoc* questions to collect general information, perception of COVID-19 experiences, and PA level. (1) Sociodemographic and COVID-19 information: this part recollected the general information of the participants (gender, age, sport type, and competition level); (2) Personal experience during COVID-19: in this section, the question to “what extent did you perceive the pandemic as stressful, challenging, controllable, and threatening?” was asked, and the athletes could respond on a 5-point Likert scale (0 = not at all, to 4 = totally), giving one response for each perception; (3) Level of PA perceived before and during the pandemic: this section evaluated the perceived daily PA level for the athletes by asking “How would you classify yourself before the pandemic, and the measures of restriction and social isolation, with respect to your daily level of physical activity?” and “how would you rate yourself today (during pandemic) in terms of your daily level of physical activity?” Participants responded to a single 5-point Likert scale ranging from 1 = very, very physically inactive to 5 = very, very physically active.

(4) The fourth section of the survey consisted of the application of the Pittsburg Sleep Quality Index questionnaire [PSQI, (29)] to measure the athletes' sleep quality after having experienced 2 years of COVID-19. The PSQI is comprised of 19 questions which are divided into seven dimensions: (1) subjective sleep quality; (2) sleep latency; (3) sleep duration; (4) habitual sleep efficiency; (5) sleep disturbance; (6) use of sleep medication; and (7) daytime dysfunction. Each dimension is rated on a 3-point scale, and the sum of all provides a global index rating from 0 to 21 points. Global scores for the PSQI > 5 are considered poor sleep quality. The use of the PSQI with athletes had been widely reported with good reliability and validity (30, 31), even during the pandemic (15), as well as with the athlete population (12, 32).

(5) The fifth, and last, section of the assessment aimed to evaluate the dietary habits at the moment of the application of the survey. The Rapid Eating and Activity Assessment for Patients (REAP-S) was used to measure this variable. The REAP-S consists of 15 items with a Likert scale of 4 points (0 = Usually/Often, to 3 = does not apply to me), which evaluates the consumption of all food groups and diet-related behaviors of the subjects (33, 34). The REAP-S score can range from 0 to 39, where higher scores indicate optimal intake of healthy foods and lower consumption of unhealthy foods. In addition, it uses one 5-point Likert scale item (1 = very willing, to 5 = not at all willing) to assess the willingness to make changes in the dietary habits of the participants. The REAP-S questionnaire has been established as a cross-cultural tool for eating behavior evaluation (35–39) and has been previously applied to athletes (34, 40) in addition to adolescents and children (41).

### Procedure

The study was carried out 2 years after the formal declaration of the SARS-CoV-2 illness (COVID-19) as a pandemic by the World Health Organization. A survey assessing the impact of COVID-19 on sleep habits, level of PA, and dietary intake was conducted over 2 months (March and April 2022). Participants responded to the survey in their native language in an online format *via* Google Forms. Informed consent to participate in the study was obtained by selecting a “check box.” A back translation method (42) was employed to translate the survey from English to Arabic, French, German, Persian, Portuguese, Spanish, and Turkish languages. For the PSQI and REAP-S, the existing translated versions were applied. The research followed the ethical recommendations for the treatment of subjects and data obtained as stated in the Declaration of Helsinki (43) and safeguarded the integrity and respected the anonymity of each participant's answers. Furthermore, the Local Ethics Committee of the Imam Khomeini International University (no. 17630) approved the study protocol.

### Statistical analysis

The statistical analysis was performed using SPSS v25.0 (SPSS Inc., Chicago, IL, USA). Data were presented as mean ± SDs and mean rank in the tables and the text. The Kolmogorov-Smirnov (KS) statistic was used to determine if a sample came from a population with a specific distribution. To analyze the differences between the social characteristics of amateur and elite athletes, we used the chi-squared test. The Mann–Whitney *U*-test and the Wilcoxon test were used to compare the differences between amateur and elite athletes before and during COVID-19. A Rho Spearman-Brown correlation analysis was employed to determine the relationships among the coronavirus pandemic experience (stressful, challenging, controllable, and threatening), sleep quality, PA level, and eating behaviors. For all the analyses, significance was set at a *p*-value of <0.05.

Finally, two independent simple moderation analyses (Model 1) were evaluated to determine the moderating roles of PA and dietary habits on the interaction between the pandemic experience and sleep quality. The PROCESS V.3.5 macro (44) extension installed in SPSS v.25 software was used. Confidence intervals (95%) were generated by bootstrapping for 10,000 samples to determine the three model effects (b1, b2, and b3). Mean ± SDs were employed to divide the PA and dietary habits levels in the moderation analysis.

## Results

**Figure 1:**
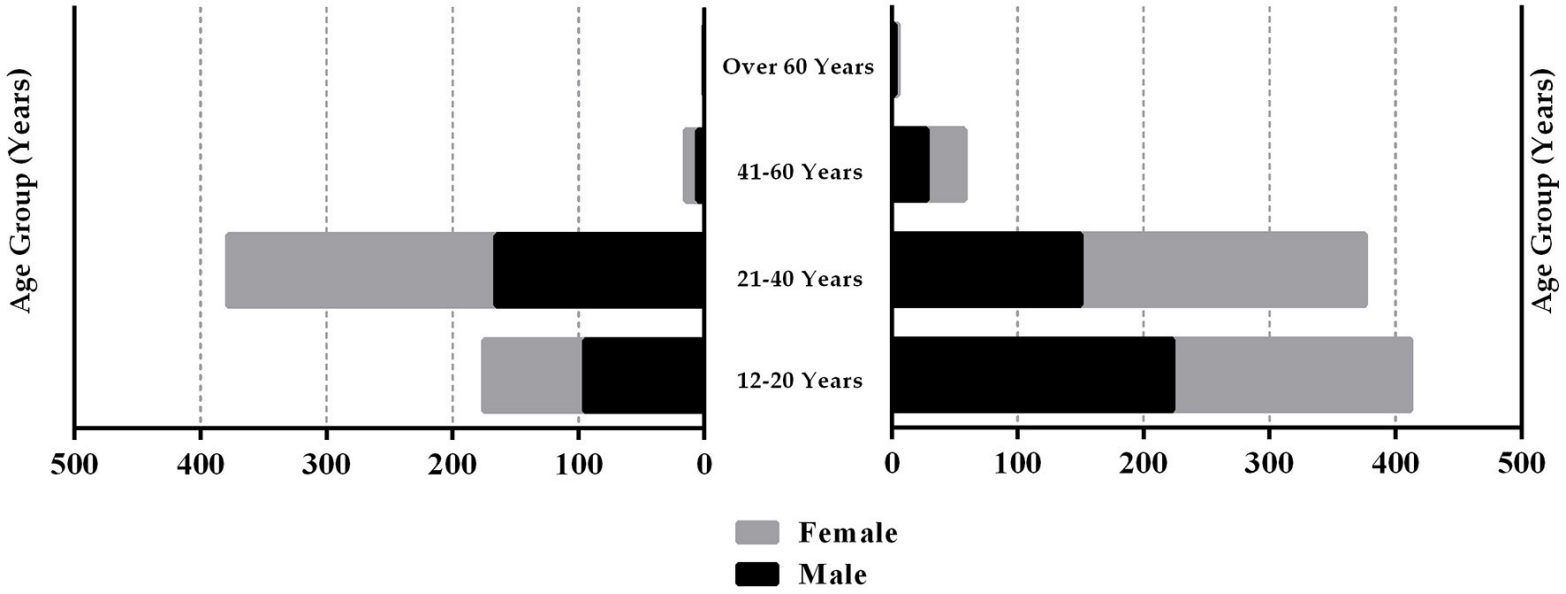
Histogram detailing the gender split and age ranges for amateur and elite athlete groups.

**Figure 2:**
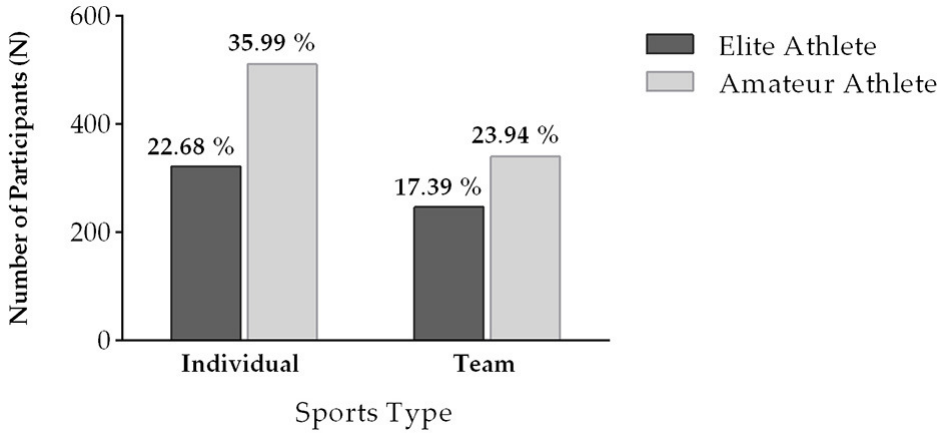
Bar chart of the type of sport practiced by the sample of elite and amateur athletes.

The demographic data of the 1,420 participants of this study were compared between the amateur and elite athlete groups using the Chi-squared test (Table 1). The histogram detailing the gender diversity and age categories for amateur and elite athlete groups is shown in Figure 1. The bar chart illustrating the type of sports played by elite and amateur athletes is shown in Figure 2. The Mann–Whitney *U*-test indicated that the PA level pre-COVID-19 was higher (*Z* = −5.94; *p* < 0.001) among elite athletes (mean = 3.79 ± 1.19) in comparison with the amateur athletes (mean = 3.43 ± 1.17). In addition, the PA level during COVID-19 was also higher (*Z* = −9.10; *p* < 0.001) among elite athletes (mean = 3.67 ± 1.13) in comparison with the amateur athletes (mean = 3.09 ± 1.17). The Wilcoxon Test indicated that the PA level pre-COVID-19 was higher than during COVID-19 in amateur (*Z* = −7.68; *p* < 0.001) and elite athletes (*Z* = −2.75; *p* = 0.006).

**Figure 3:**
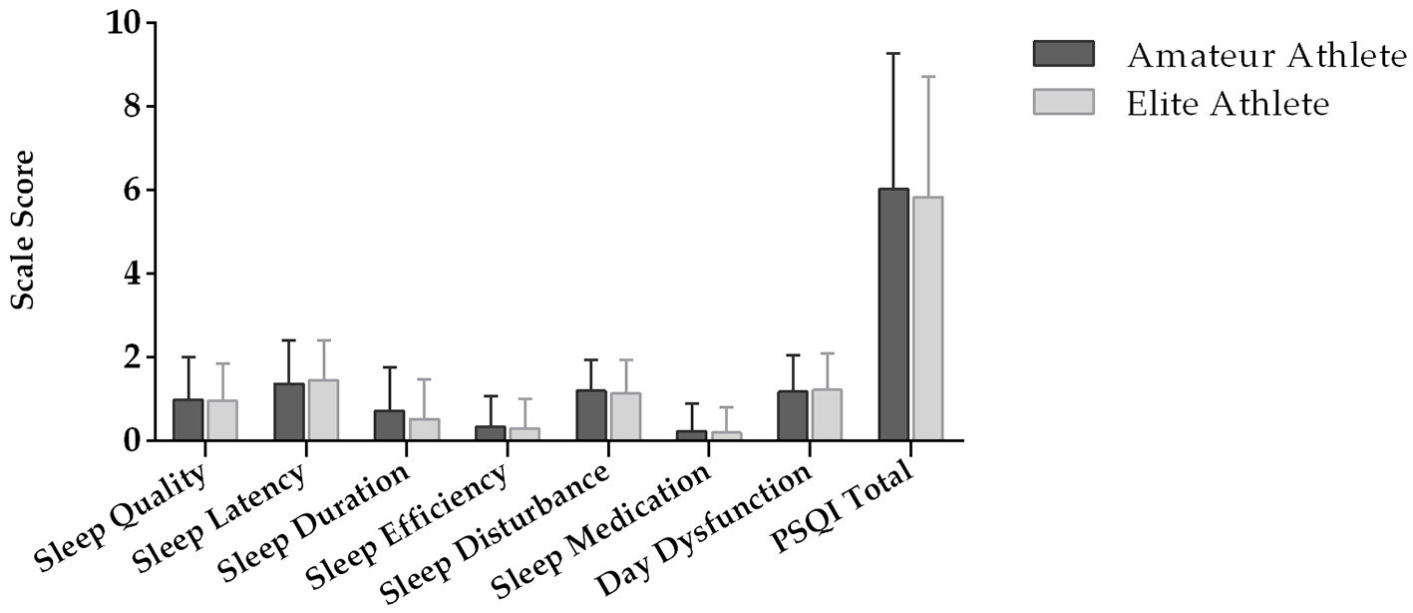
Sleep quality subscales and total sleep quality score index (PSQI). ^*^Difference between groups at *p* < 0.05.

The analysis of the intergroup differences (elite vs. amateur) for sleep quality, dietary habits, and COVID-19 experience can be observed in Table 2. On sleep quality, the results showed no significant difference (*p* > 0.05) in the PSQI total scale as well as in all sleep quality subscales (Figure 3) except sleep duration (*p* < 0.001). With respect to dietary habits, a significant difference in the mean rank of REAP between elite and amateur athletes (*p* < 0.05) was observed. The analysis of experience perception during COVID-19 revealed that elite athletes perceived a controllable experience significantly more than amateur athletes (*p* < 0.05).

**Table 2:** Results of the Mann–Whitney test for the PSQI, REAP-S, and personal experience during the COVID-19 pandemic in amateur and elite athletes.

	**Amateur(*****n*** = **851)**		**Elite (*****n*** = **569)**		***Z* (*p*-value)**
	**Mean**	±**SD**	**Mean**	**SD**	
PSQI total scale	6.03	3.25	5.84	2.89	−0.76
REAP-S	24.69	7.48	23.69	7.44	−2.45^*^
**Personal experience**					
Stressful	1.49	1.38	1.51	1.38	−0.22
Challenging	1.57	1.43	1.65	1.40	−1.04
Controllable	1.84	1.37	2.02	1.33	−2.32^*^
Threatening	1.56	1.47	1.47	1.35	−0.69

PSQI Total Scale, Sum of the punctuations by the Pittsburg Sleep Quality Index; REAP-S, Short version of the Rapid Eating Assessment of Patients.

^*^p < 0.05.

No differences were observed in the other perceived experiences during the pandemic; however, many significant (*p* < 0.05) correlations could be observed between the variables (Table 3). The stressful personal experience of COVID-19 had a positive relationship with sleep quality (higher scores in the PSQI represent a lower sleep quality) and a negative relationship with the PA level. The perception of a challenging experience had positive correlations with sleep quality and dietary habits and a negative relationship with PA level. The perception of the COVID-19 experience as controllable and threatening also correlated negatively with sleep quality and PA level. In addition, the PA level showed a negative correlation with sleep quality and a positive correlation with eating behaviors.

**Table 3:** Correlations between the personal COVID-19 experience, PSQI, physical activity, and REAP-S in athletes.

	**Stressful**	**Challenging**	**Controllable**	**Threatening**	**PSQI total**	**PA**
Challenging	0.78^**^	–				
Controllable	0.33^**^	0.37^**^	–			
Threatening	^**^0.70	^**^0.72	^**^0.36	–		
PSQI total	0.08^**^	0.09^**^	−0.08^**^	0.06	–	
PA	−0.08^**^	−0.07^*^	−0.03	−0.11^**^	−0.06^**^	–
REAP-s	0.05	0.06^*^	−0.01	0.03	−0.02	0.09^**^

PSQI total, the summed punctuations of the Pittsburg Sleep Quality Index; PA, physical activity level; REAP-S, the Rapid Eating and Activity Assessment for Patients.

^*^p < 0.05.

^**^p < 0.01.

**Figure 4:**
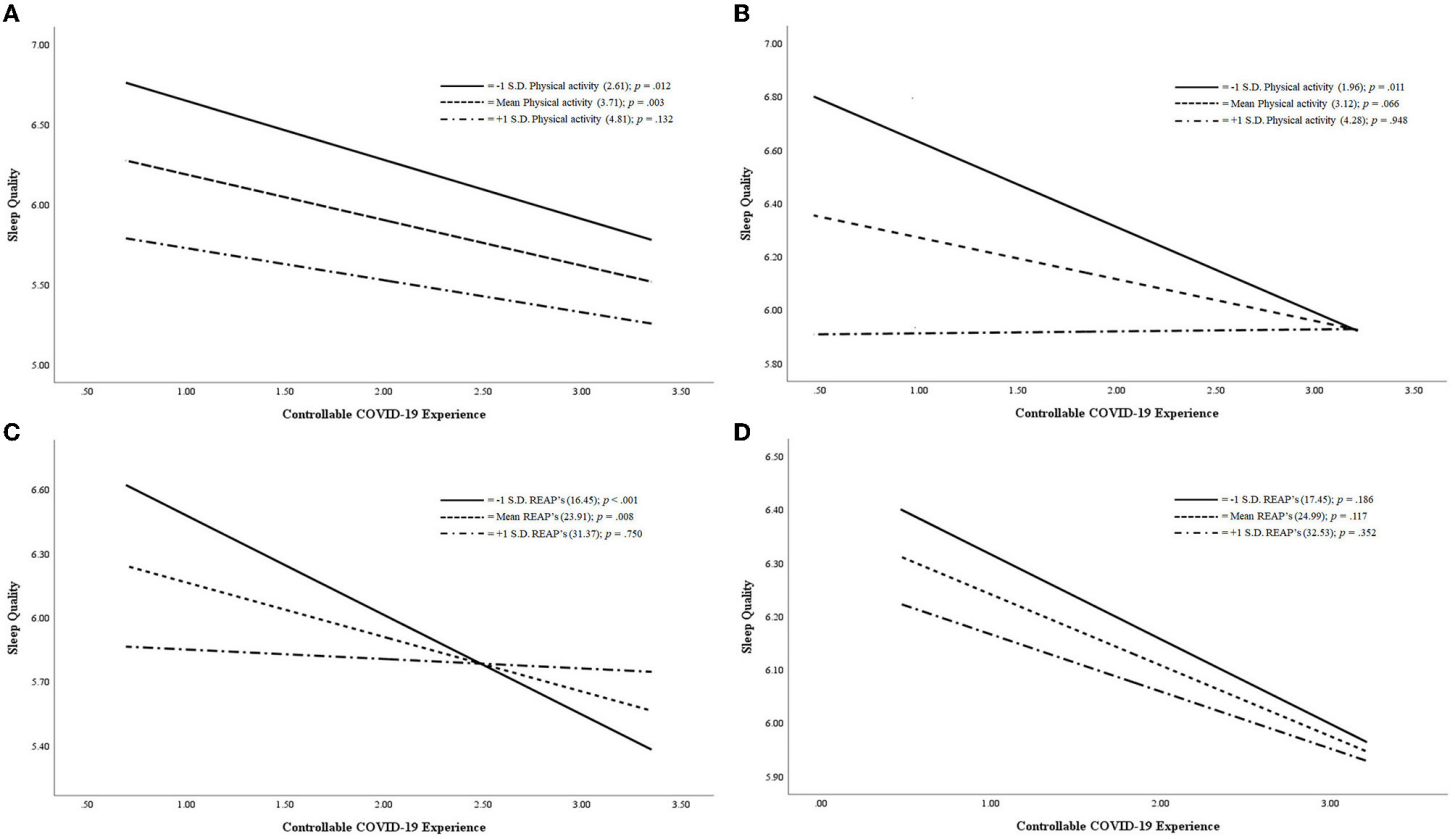
The moderating effect of physical activity level **(A, B)** and dietary habits **(C, D)** on sleep quality by controllable COVID-19 experience in elite and amateur athletes.

The (separate) moderation analysis (Figure 4) of PA and dietary habits for elite and amateur athletes (Table 4) showed one statistically significant and one trend significant model. The amateur athletes [*F*_(3,777)_ = 3.05; *p* = 0.028], with PA level (W), had a trend moderating effect on the interaction between COVID-19 personal experience as controllable (X) and sleep quality (Y). The regression coefficient exposed that the effect of PA on sleep quality was negative and statistically significant (*b*_[PA]_ = −0.450; *p* = 0.010), demonstrating that, in amateur athletes, the level of PA during the pandemic had an influence on sleep quality (lower index in PSQI Total). In addition, the effect of the independent variable on the dependent variable was negative and statistically significant (*b*_[ControllableCOVID − 19Experience]_ = −0.596; *p* = 0.019), indicating that amateur athletes with higher controllable COVID-19 experience had greater sleep quality (lower index in PSQI Total). A trend-significant interaction effect of both variables (*b*_[ControllableCOVID − 19ExperienceXPA]_ = 0.141; *p* = 0.057) on sleep quality was also found, proving that amateur athletes who had moderate and high PA levels during the pandemic had better sleep quality regardless of their perception of a controllable pandemic situation. However, it was found that dietary habits (W) had a moderating effect [elite athletes: *F*_(3,506)_ = 4.47, *p* = 0.004] on the interaction between COVID-19 personal experience as controllable (X) and sleep quality (Y). The regression analysis showed that dietary habits had a statistically significant negative effect on sleep quality (*b*_[DietaryHabits]_ = −0.071; *p* = 0.015), denoting that elite athletes with better dietary habits had a lower PSQI index (better sleep quality). Regarding the controllable COVID-19 experience effect on sleep quality, it showed a negative and statistically significant regression coefficient (*b*_[ControllableCOVID − 19Experience]_ = −0.933; *p* = 0.003), meaning that, when elite athletes perceived the COVID-19 situation to be more controllable, they had a better sleep quality (lower PSQI index). Finally, the interaction effect of both variables on sleep quality was positive and statistically significant (*b*_[ControllableCOVID − 19ExperienceXDietaryHabits]_ = 0.028; *p* = 0.028). This result can be understood as the impact that better dietary habits have on sleep quality in elite athletes irrespective of their perception of the pandemic as controllable.

**Table 4:** Moderation analysis coefficients of physical activity and dietary habits.

		**Sleep quality (** ***Y*** **)**			
		**Elite athletes**		**Amateur athletes**	
		**Coefficient (SD)**	**95% CI**	**Coefficient (SD)**	**95% CI**
(*X*) Controllable COVID-19 experience	*b* ^1^	−0.570 (0.368)	−1.293, 0.153	−0.596 (0.254)^*^	−1.095, −0.097
(*W*) Physical activity	*b* ^2^	−0.495 (0.222)^*^	−0.930, −0.060	−0.450 (0.176)^**^	−0.795, −0.105
X ^*^ W	*b* ^3^	0.077 (0.093)	−0.106, 0.259	0.141 (0.074)^+^	−0.004, 0.286
Constant		8.310 (0.882)^***^	6.577, 10.044	7.834 (0.603)^***^	6.650, 9.017
		*R*^2^ = 0.032; *F*_(3,506)_ = 5.538^***^		*R*^2^ = 0.012; *F*_(3,777)_ = 3.049^*^	
(*X*) Controllable COVID-19 experience	*b* ^1^	−0.933 (0.317)^**^	−1.557, −0.309	−0.219 (0.287)	−0.782, 0.345
(*W*) Dietary habits	*b* ^2^	−0.071 (0.029)^*^	−0.128, −0.013	−0.013 (0.024)	−0.060, 0.033
X ^*^ W	*b* ^3^	0.028 (0.013)^*^	0.003, 0.054	0.003 (0.011)	−0.018, 0.024
Constant		8.106 (0.735)^***^	6.662, 9.594	6.709 (0.622)^***^	5.489, 7.929
		*R^2^* = 0.026; *F*_(3,506)_ = 4.468^**^		*R*^2^ = 0.004; *F*_(3,777)_ = 0.938	

^+^p < 0.10.

^*^p < 0.05.

^**^p < 0.01.

^***^p < 0.001.

## Discussion

This study examined the influence that levels of PA and dietary habits had on the sleep quality and perception of experiences of elite and amateur athletes during COVID-19. The results of the study focused on the moderating role that PA in amateur athletes and dietary habits in elite athletes had on the effect of perceiving a controllable COVID-19 experience over sleep quality as measured by the PSQI.

### The moderating effect of physical activity and dietary habits

Both moderation models that resulted in a significant interaction (dietary habits; *p* < 0.05) and trend-significant interaction (PA; *p* = 0.057) demonstrated that low levels of PA (in amateur athletes), as well as less proper dietary habits (in elite athletes), did not favorably regulate sleep quality. However, moderate and high levels of PA and better dietary habits facilitated improved sleep quality regardless of how controllable or not the athletes perceived the situation resulting from the COVID-19 pandemic to be. Previous research has pointed out the relationship between decreased exercise frequency with sleep problems and stress (14, 45), showing that high levels of PA contribute to the management of negative experiences and cognitive anxiety over suspicions of COVID-19 infection (27). In addition, the study by Romdhani et al. (13) showed that the influence of higher levels of training during the lockdown period was associated with a better sleep quality index.

Similarly, there is consensus in the literature on the influence of diet has on sleep quality in athletes (46, 47). Practices and modifications in dietary habits can alter sleep patterns and sleep quality in athletes, as reported by Lipert et al. (48) in athletes who adhere to Islam's code for the practice of the daytime fast during Ramadan. Similarly, the unregulated use of supplements such as caffeine or substances such as alcohol is associated with sleep quality disturbances (49).

According to previous research, elite athletes are accustomed to following a stricter diet than amateurs (49, 50). The influence of dietary habits on the sleep quality of elite athletes can be explained by the need of this population to maintain their fitness during the pandemic period through eating behaviors familiar to those they had before. Previous studies have reported alterations in the sleep quality of athletes due to modifications in their dietary habits (48, 51). Consequently, elite athletes who change their dietary habits to consume more carbohydrates and less protein or increase their caffeine or alcohol intake would be more susceptible to suffering from sleep disturbances as reported in the literature (46, 52).

### Sleep quality

The results of the comparison between both groups of athletes did not show statistically significant differences in the global index of sleep quality as both presented a score (>5) that was associated with poor sleep quality (29). This similarity coincides with the study by Jemal et al. (53), which suggests the need to educate athletes on sleep hygiene, regardless of the level at which they compete. During the pandemic, different sleep disturbances or disorders generated by changes in athletes' schedules (13, 45), alterations in dietary habits (15), and increased stress levels (14) have been widely reported. These reports have emphasized the need for athletes to regulate their sleep periods to improve their quality since sleep is one of the main tools to achieve optimal recovery (49). Not addressing these factors led to an increase in daytime sleepiness (12) and a decrease in sports performance (48).

Sleep quality is an essential part of the recovery process for athletes. In our study, the mean global index score of PSQI was close to 6 for both groups, which is cataloged as poor quality. Many studies have reported similar values of sleep quality measured with the PSQI in elite and amateur athletes during the pandemic (13, 45, 46). In the present study, the component of sleep duration of the PSQI was statistically significant, being better for elite athletes. Previous research has pointed out the differences between elite and amateur athletes in sleep duration (53). Even when the number of hours in bed and sleep duration increased during the pandemic (13, 14, 45, 54), the sleep quality got worse due to the changes in daily life routines and habits of the athletes. According to the results of this study, improving sleep quality will benefit the physical condition and health of the athletes (55, 56).

The Global Index of PSQI shows a negative and significant correlation with the PA level during COVID-19, as well as with the perception of controllable experience during the pandemic. Both results can be interpreted as expected behaviors in the relationship between variables. A higher score in PSQI is associated with worse sleep quality; therefore, when PA and controllable COVID-19 experience were high, a lower score in the global PSQI was observed. However, a positive association (*p* < 0.05) was identified between the global index of sleep quality and the perceptions of the pandemic being stressful and challenging. Similar results have been noted in previous studies (13, 14, 54). Thus, variables such as an adequate level of PA or having a positive perception of the problem that athletes are faced with are protective factors to promote good sleep quality.

### Physical activity

The differences observed between elite and amateur athletes in PA levels confirmed that, even though both groups decreased their level of PA during the pandemic compared to pre-pandemic levels, elite athletes continued to exercise more. These results are consistent with previous studies indicating a significant decrease in PA levels during COVID-19 (57), with differences between elite and amateur athletes in their levels of PA during the pandemic period (11, 58, 59). According to the literature, even in the face of adversity, higher-performing (elite) athletes tended to diligently continue their training routine compared to lower-level (amateur) athletes (15, 58).

Moreover, the evidence obtained during the pandemic period points out that elite or higher-level athletes had more support from their physical trainers and coaches (5, 57) and access to necessary equipment (11, 58), and, additionally, their experiences allowed them to have more certainty that they were performing the training sessions adequately (7, 45, 59). Notwithstanding the foregoing, the PA levels were lower during the COVID-19 pandemic period than before for both elite and amateur athletes, even when the data for the present study were collected 2 years after the pandemic was declared. This result may be due to the period when the data were collected, as the pandemic was not over and continued social restrictions and coronavirus-related anxiety may have limited the return to normal sports practice (25).

In addition, the variable PA showed a positive and significant correlation with REAP-S. Whereas, PA had a negative association (*p* < 0.05) with the perception of COVID-19 as stressful, challenging, and threatening. A few previous studies have reported a positive relationship between PA and eating behaviors, both during the pandemic period (1, 60, 61) and before the pandemic (50, 52, 62). Furthermore, in the same vein, more than sleep quality, a better PA level was associated with a less negative experience of the problems that athletes face (5, 27, 61, 63).

### Dietary habits

The analysis of the dietary habits of athletes during the pandemic showed that amateur athletes presented better eating behaviors than elite athletes. This phenomenon is contrary to most of the findings reported in the literature (61, 64). It is possible that elite athletes follow a stricter dietary regimen which is linked to their specific sports performance goals. For this reason, based on what has been pointed out in some research, the break in normality due to the pandemic and the cancellation and changes in the competition schedule could have resulted in the relaxation and adaptation to the demands of elite athletes (20, 60).

Despite the differences observed between elite and amateur athletes, it is possible that both have adequate dietary habits (40, 65). This result is important because some studies have reported that athletes tend to have poor eating behaviors (50, 66), suggesting that athletes must be educated and receive support from a professional nutritionist. Nevertheless, in their study, Newbury et al. (60) highlighted the good eating habits of swimmers who adjusted their diet during the pandemic and the most restrictive lockdown periods that followed, illustrating the relevance of promoting nutritional education in athletes to maintain proper physical fitness to perform well and for healthy wellbeing.

### Athletes' COVID-19 pandemic experience

The last variable examined in the present study focused on analyzing the perception of personal experience during COVID-19. Only the perception of the COVID-19 experiences as controllable showed significant differences between groups, with those being higher among elite athletes. In this sense, both prior to and during the pandemic, several investigations have pointed out how resilience and continuous exposure to stress among elite athletes allow them to develop more effective coping strategies (23), as well as to perceive adversities as challenges that they can cope with adequately (5, 22, 58).

The perception of the pandemic as stressful, challenging, or threatening was no different for the athletes regardless of their expertise level. These were three negative expressions that participants were asked to identify in this study how they experienced the situation during the COVID-19 pandemic. Similar studies reported how social changes emerged because of the social restrictions due to COVID-19 and impacted the athletes' mood and wellbeing (21, 24, 57, 63). As a positive expression, the athletes were asked whether their perception of their experience during the COVID-19 pandemic was controllable. It is well-known that athletes learn and develop how to deal with problems in a better form than other people (26), particularly elite athletes more so with their enhanced experiences and abilities (10, 23). Therefore, the relevance of these results is that they emphasize the fact that assessing the athletes' perception of their experience is necessary for a holistic understanding of how they lived during the pandemic.

### Study limitations and strengths

This study is part of a research project whose main strength is the data collected from 14 countries spread across four continents. In addition, the respondents being drawn from several sports fields and competition levels (elite and amateur) have made the results of this study relevant to the scientific community. The results presented in this study allow the reinforcement of the relevance of PA for both groups of athletes but mostly for amateur-level athletes. The results also reiterate the need to maintain or improve dietary habits among the athletic community, which are very important, primarily for elite athletes to improve their sleep quality. Nevertheless, this study had some limitations. Psychological factors, stress levels, or mental illness diagnoses such as anxiety or depression were not explored within the context of coping mechanisms. Additionally, differences in a few of the sociodemographic factors such as marital status, financial security, or mental health support between the sporting levels, which could play a confounding role in the results of the study, are worth exploring. Furthermore, the time period when the study was carried out could have altered the answers provided by the athletes; most of the countries where data was collected were under a new normal following the pandemic.

## Conclusion

Elite athletes had different lifestyle behaviors compared to amateurs during the COVID-19 lockdown. Although there was no difference in the sleep quality of elite athletes and amateurs, the dietary habits of amateurs were better than those of elite athletes, but a superior PA level was observed in elite athletes during the pandemic. Furthermore, the relevance of maintaining high levels of PA for amateurs and good quality dietary habits for elite athletes was noted by the moderating effect that both variables had on the influence of the controllable experience during the pandemic on sleep quality. Thus, the positive influence that the perception of the pandemic experience as controllable had over sleep quality in athletes was improved by high PA levels in amateur athletes, as well as by good nutritional regimens in elite athletes.

## Data availability statement

The original contributions presented in the study are included in the article/supplementary material, further inquiries can be directed to the corresponding author.

## Ethics statement

The studies involving human participants were reviewed and approved by the research followed the ethical recommendations for the treatment of subjects and the data obtained as stated in the declaration of Helsinki (World Medical Association, 2013) safeguarded the integrity and respected the anonymity of each participant's answers. Furthermore, the Local Ethics Committee of the Imam Khomeini International University (no. 17630) approved the study protocol. The patients/participants provided their written informed consent to participate in this study.

## Author contributions

All authors listed have made a substantial, direct, and intellectual contribution to the work and approved it for publication.
